# Changes in the Percentage of Patients Treated for Cancer Before and After the SARS-CoV-2 Epidemic: A Retrospective Observational Study

**DOI:** 10.14789/ejmj.JMJ24-0015-OA

**Published:** 2024-12-31

**Authors:** YUKARI MAEHARA, KAZUTOSHI FUJIBAYASHI, RYOHEI KUWATSURU, HIROYUKI DAIDA, SHIGEKI AOKI

**Affiliations:** 1 Department of Data Science Course, Graduate School of Medicine, Juntendo University, Tokyo, Japan; 1 Department of Data Science Course, Graduate School of Medicine, Juntendo University, Tokyo, Japan; 2 Astellas Pharma incorporated, Tokyo, Japan; 2 Astellas Pharma incorporated, Tokyo, Japan; 3 Medical Technology Innovation Center, Juntendo University, Tokyo, Japan; 3 Medical Technology Innovation Center, Juntendo University, Tokyo, Japan; 4 Department of General Medicine, Juntendo University Faculty of Medicine, Tokyo, Japan; 4 Department of General Medicine, Juntendo University Faculty of Medicine, Tokyo, Japan; 5 Department of Radiology, School of Medicine and Graduate School of Medicine, Juntendo University, Tokyo, Japan; 5 Department of Radiology, School of Medicine and Graduate School of Medicine, Juntendo University, Tokyo, Japan; 6 Faculty of Health Science, Juntendo University, Juntendo University Graduate School of Medicine, Tokyo, Japan; 6 Faculty of Health Science, Juntendo University, Juntendo University Graduate School of Medicine, Tokyo, Japan

**Keywords:** SARS-CoV-2, pandemic, regular outpatients, cancer

## Abstract

**Objectives:**

After the severe acute respiratory syndrome corona virus 2 (SARS-CoV-2) outbreak, a state of emergency was imposed to stop the spread of infection, resulting in restrictions on routine medical examinations. As a result, there has been a decline in cancer screening and detection. However, it is uncertain how many more cancer cases among routine outpatients have been detected recently.

**Methods:**

We retrospectively identified regular outpatients with no history of cancer treatment at the Juntendo University Hospital. The difference in the percentage of these patients who initiated cancer treatment within the following year, before and after the SARS-CoV-2 pandemic was analyzed.

**Results:**

A total of 33,417, 32,579, and 30,303 regular outpatients with no history of cancer treatment were identified for fiscal years 2018, 2019, and 2020, respectively. The percentage of these patients with new cancer treatment within the following fiscal year was 454 (1.36%) for 2018, 440 (1.35%) for 2019, and 416 (1.37%) for 2021. There was no statistically significant difference in the percentage of patients initiating cancer treatments before and after the SARS-CoV-2 pandemic (2018 vs. 2020, 2019 vs. 2020, respectively P = 0.88, 0.81) among patients who regularly visited outpatients at our hospital.

**Conclusions:**

The SARS-CoV-2 pandemic had no effect on the percentage of regular outpatients newly treated for cancer.

## Introduction

The first case of the severe acute respiratory syndrome corona virus 2 (SARS-CoV-2) was discovered in Japan in January 2020, after it originally emerged in Wuhan, China, in November 2019. From April 7 to May 25 of 2019, due to a rise in the number of infected patients, Japan declared its first state of emergency to stop the spread of SARS- CoV-2. Since then, as we write the manuscript, a total of four states of emergency have been issued. Our social interactions have changed as a result of so many states of emergency having been declared.

The first state of emergency mandated that patients refrains from visiting to medical institutions and consequently, their health examinations were postponed. According to the Japan Cancer Society, approximately 2,100 tumors could have gone undetected in 2020 due to a reduction in cancer screenings compared to 2019^1-4)^. Numerous reports indicate that cancer detection is delayed not just in Japan but also in other countries^5-7)^.

However, in clinical practice, advanced imaging screening, computed tomography scans, and positron emission tomography-CT scans can opportunistically identify early-stage cancer^8-10)^. Furthermore, regular blood sampling of the patient aids in the early detection of unexpected abnormalities. Diabetes is one of the chronic diseases requiring regular medical visits. For patients whose treatment has changed or needs adjustment, or those who have not achieved their glycemic goals, quarterly HbA1c testing is recommended^11)^. In primary care, the perception of the physician’s health as “fairly poor” and consultations involving changes in management were most strongly associated with shorter follow-up intervals, with the most common intervals being 12 weeks and 16 weeks^12)^. In Japan, many patients with chronic illnesses regularly visit medical institutions every 1-3 months. Additionally, it is recommended to undergo blood tests every few months. Consequently, these regular visits to medical facilities have helped the early detect of cancer, in scenarios outside of routine checkups. As a result, we anticipated that restricting regular patient visits may interfere with the incidental detection of cancer in the clinical setting, particularly among patients who had been visiting their doctor regularly, potentially leading to increased mortality rate and disease progression^13-15)^.

To the best of our knowledge, no studies have been conducted to investigate the effects of the SARS-CoV-2 pandemic on number of patients newly treated for cancer in patients receiving regular checkups. As a result, we performed this study to clarify whether there was a difference in the rate for new cancer treatment before and after the SARS-CoV-2 pandemic in patients who regularly visit hospitals for chronic diseases.

## Materials and Methods

### Study design and duration

This is a retrospective repeated cross-sectional study that examines the cancer detection rates of patients who were regular outpatients at Juntendo University Hospital from April 2017 to April 2021.

### Data collection and handling

Patients who visited Juntendo University Hospital between April 2017 and March 2021 and who were prescribed medication at least once every three months by the same department were defined as “regular outpatients” for analysis. We defined “cancer patients” as those with an ICD-10 code C00-C97 or D00-D09 or who have a history of pharmacotherapy or surgical procedures for cancer treatment. All patients were at least 18 years of age.

### Statistical analyses

Cancer detection rates for each group were compared using the chi-square test. All computations were carried out using the statistical software program JMP, version PRO 16 (SAS Institute Inc., Cary, NC, USA); a P-value of <0.05 was considered statistically significant.

## Results

To determine whether there was a difference in the number of cancer diagnoses among patients who visited regular outpatient clinics before and after the SARS-CoV-2 epidemic, we compared patients who visited regular outpatient clinics from April 2017 to March 2019 (control groups) to patients who visited regular outpatient clinics from April 2020 to March 2021 (comparator groups).

**Figure 1:**
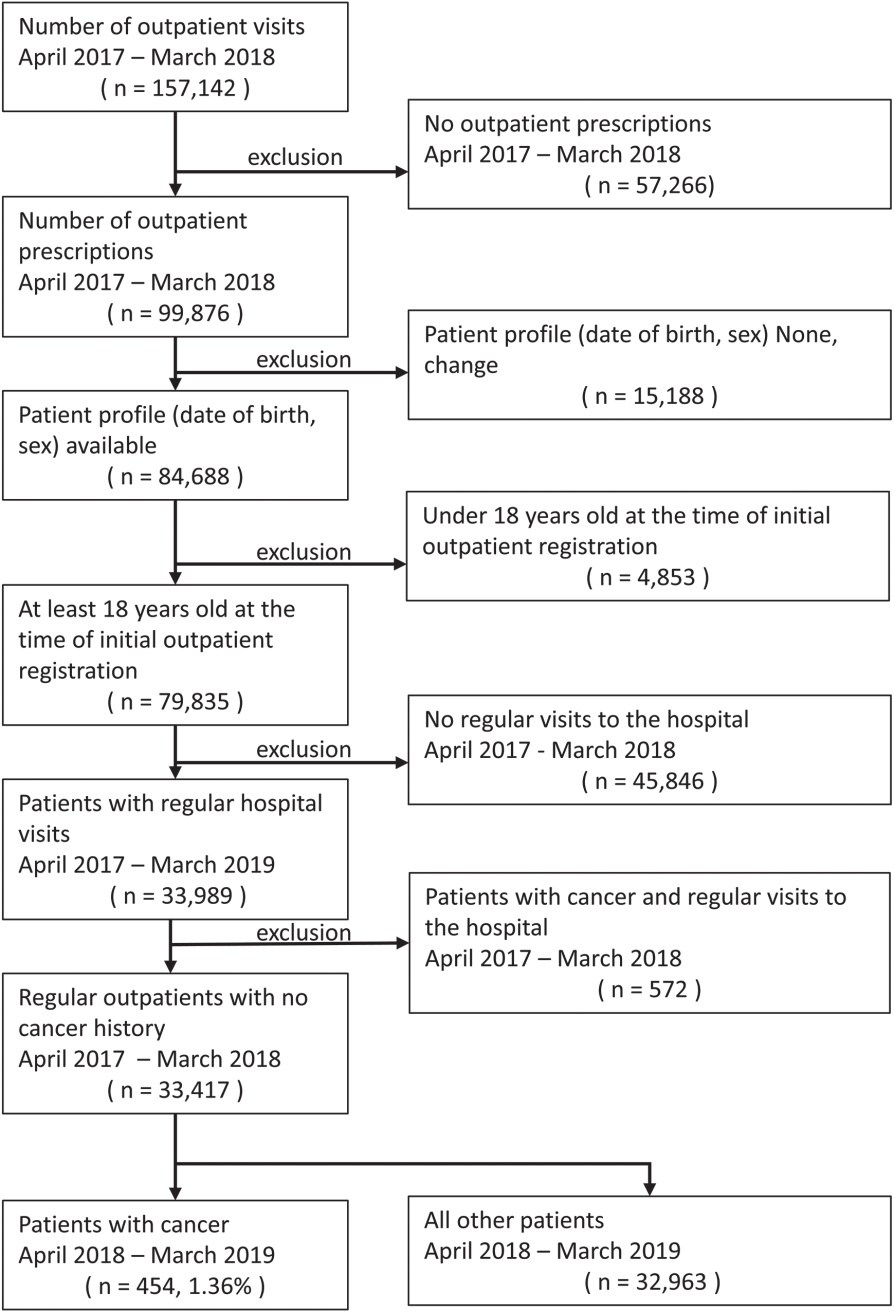
Cancer detection flow control group 1

**Figure 2:**
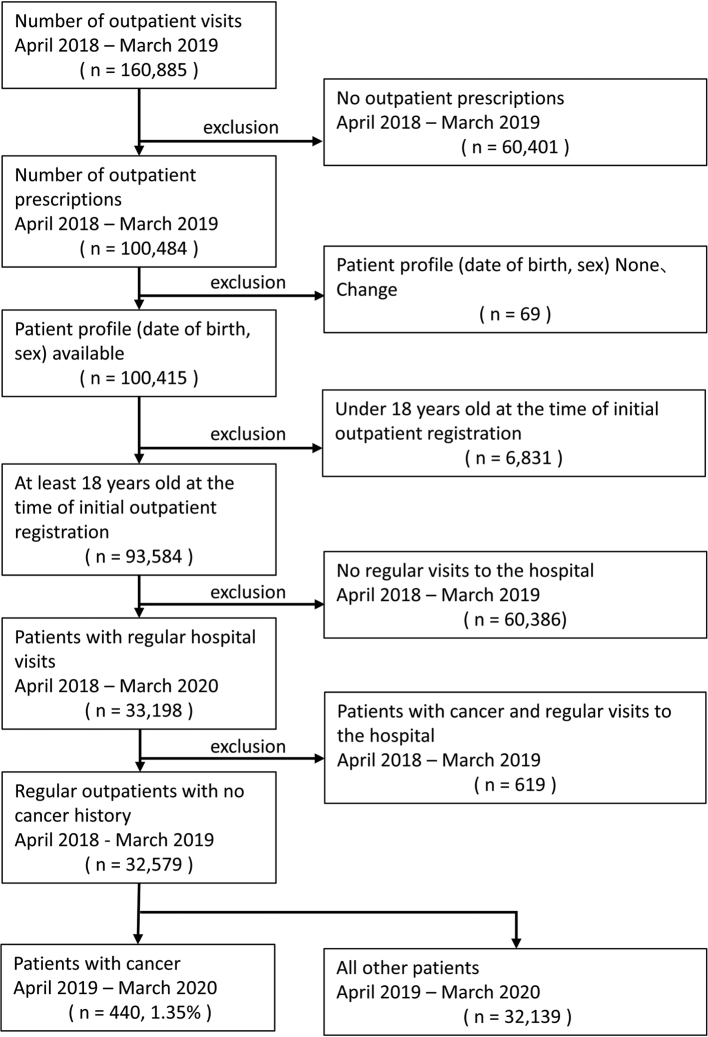
Cancer detection flow control group 2

**Figure 3:**
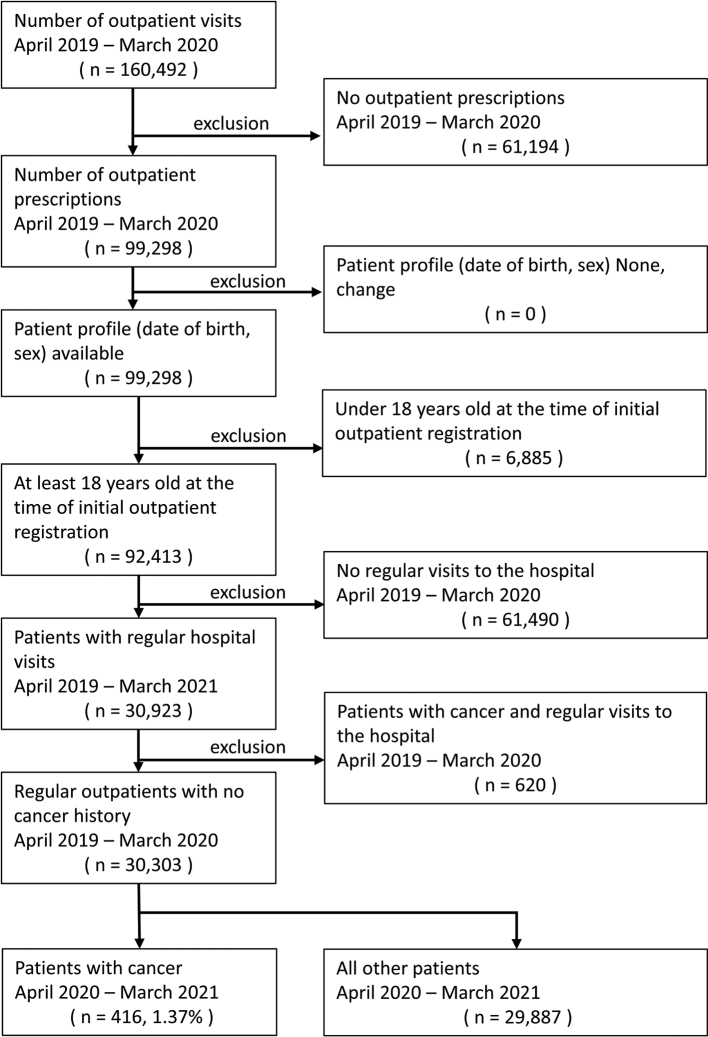
Cancer detection flow control group 3

There were 454 (1.36%), 440 (1.35%), and 416 (1.37%) cancer diagnoses from April to March of 2018 (Figure 1), 2019 (Figure 2), and 2020 (Figure 3), respectively (fiscal years 2018, 2019, and 2020).

There was no difference in the cancer detection rates between groups, with P values of 0.88 and 0.81 (Table 1). In each group, the top three cancer diagnoses were the same. The first three were malignant neoplasms of the bronchus and lung, malignant neoplasms of the prostate, and malignant neoplasms of the breast. Malignant neoplasms of the stomach did not, however, rank among the top five cancer detections in FY 2020, despite ranking fourth in FY 2018 and FY 2019. (Tables 2-4).

**Table 1:** Comparison of number of patient with new cancer treatment among regular outpatients without cancer history in 2018, 2019, and 2020. Chi-square test was used to compare the number of patients with cancer treatment in the following year

	Regular outpatients with no cancer history		
	No cancer treatment in the following year (n)	Cancer treatment in the following year (n)	P value
2018	32,963	454	0.88^＊^
2019	32,139	440	0.81^†^
2020	29,887	416	

^＊^2018 vs. 2020, ^†^2019 vs. 2020

**Table 2:** Top 10 new cancer detected based on ICD-10 code for the 2018 group

ICD-10 Version: 2019	Number of patients
Malignant neoplasm of bronchus and lung	82
Malignant neoplasm of prostate	80
Malignant neoplasm of breast	60
Malignant neoplasm of stomach	40
Malignant neoplasm of colon	34
Malignant neoplasm of liver and intrahepatic bile ducts	33
Malignant neoplasm of meninges	29
Secondary malignant neoplasm of other and unspecified sites	27
Malignant neoplasm of kidney, except renal pelvis	20
Malignant neoplasm of rectum	19
Secondary malignant neoplasm of respiratory and digestive organs	19

**Table 3:** Top 10 new cancer detected based on ICD-10 codes for the 2019 group

ICD-10 Version: 2019	Number of patients
Malignant neoplasm of bronchus and lung	72
Malignant neoplasm of prostate	63
Malignant neoplasm of breast	59
Malignant neoplasm of stomach	48
Malignant neoplasm of liver and intrahepatic bile ducts	46
Malignant neoplasm of colon	31
Malignant neoplasm of bladder	30
Secondary malignant neoplasm of other and unspecified sites	25
Secondary malignant neoplasm of respiratory and digestive organs	16
Malignant neoplasm of esophagus	15

**Table 4:** Top 10 new cancer detected based on ICD-10 codes for the 2020 group

ICD-10 Version: 2019	Number of patients
Malignant neoplasm of bronchus and lung	69
Malignant neoplasm of prostate	54
Malignant neoplasm of breast	52
Malignant neoplasm of liver and intrahepatic bile ducts	47
Secondary malignant neoplasm of respiratory and digestive organs	38
Malignant neoplasm of colon	37
Malignant neoplasm of stomach	36
Secondary malignant neoplasm of other and unspecified sites	30
Secondary malignant neoplasm of other and unspecified sites	28
Malignant neoplasm of esophagus	18

## Discussion

In this investigation, there was no change in the percentage of new cancer treatment before and after the SARS-CoV-2 pandemic in regular outpatients. Furthermore, the fact that the top three cancer detections did not change before and after the SARS-CoV-2 pandemic indicates that no significant cancers were missed due to interruptions in routine medical checkups. This outcome was not what we had anticipated. This may be because this study defined regular patients as those who visited Juntendo University Hospital at least once a quarter. In generally, new cancers are likely to be naturally diagnosed during regular outpatients or periodic health examinations such as comprehensive medical check-ups. Despite the spread of SARS-CoV-2, patients who visited Juntendo University Hospital regularly received consistent medical management and necessary examinations, which may have contributed to the maintenance of the overall number of cancer diagnoses. We speculate that malignancies were found incidentally when patients had regular outpatient visits, which is why the percentage of patients with new cancer treatment among regular outpatient did not decline.

The SARS-CoV-2 pandemic has significantly decreased cancer screening rates worldwide. In 2022, Lee et al. performed an annual cross-sectional study using data from the Korean National Cancer Screening Survey and reported that the screening rates for the four major cancers investigated (stomach, colorectal, breast, and cervical cancer) were significantly lower. Interestingly, this research also reported screening was significantly lower among those with no history of chronic disease^16)^. However, according to the findings of our study, people with chronic diseases may have not missed their routine visits as suggested, which may partly explain why cancer detection rates have not fallen. Our new theory is supported by the findings of this paper.

However, our data also show results suggestive of the impact of the SARS-CoV-2 pandemic. The top three categories remained the same, but the number of gastric cancers diagnosed in control groups 1 and 2 decreased from more than 40 per year in control groups 1 and 2 to less than 40 per year in the comparator groups. The number of malignancies detected did not significantly change over the year, but when the state of emergency was declared, endoscopies and operations were only permitted in emergencies. This restriction on endoscopy in non-urgent cases may have reduced gastric cancer detections^17)^.

There are several limitations to this study. The first is that this study is not generalizable because it is based only on Juntendo University Hospital. The results of this study may also be influenced by selection bias. Second, we did not directly check electronic medical records for cancer diagnosis. In this study, cancer is defined in terms of the disease’s nomenclature, its surgical or medical treatments, and its medication treatments. Thus, it includes the possibility of variations in the diagnostic names of ICD-10 codes. And only number of cancer diagnosis was collected also number of diagnostic examinations, which are CT, GIF, CF, etc, were not considered in the analysis. Additionally, the history of undergoing regular health check-ups, such as human dry dock, is also not collected in this study. These limitations may affect the accuracy and reliability of the study’s results. Third, we did not consider cancers found before the observation period. Cases considered to be cancer in this research may include cases of recurrence/metastasis of cancer discovered before the observation period. Even throughout the epidemic, regular examinations and follow-up care of cancer patients would have been conducted. Fourth, the three major treatments for cancer are pharmacotherapy, surgery, and radiotherapy, but this study did not investigate radiotherapy. Future research directions include additional analyses based on information from more medical institutions, removal of fluctuations in ICD-10 codes, and exclusion of recurrent and metastatic malignancies from new cancer detections.

## Conclusions

In this study, there was no difference in the percentage of regular outpatients diagnosed with cancer before and after the novel coronavirus pandemic.

## Funding

This research received no external funding.

## Author contributions

Conceptualization, YM, KF, RK, HD and SA; methodology, YM and KF; validation, YM and KF; formal analysis, YM and KF; investigation, YM and KF; data curation, YM and KF; writing-original draft preparation, YM and KF; writing-review and editing, YM, KF, RK, HD and SA; supervision, SA; project administration, KF All authors have read and agreed to the published version of the manuscript.

## Conflicts of interest statement

The authors declare no conflict of interest. Ryohei Kuwatsuru, one of the Editorial Board members of JMJ was not involved in the peer review or decision-making process for this paper.

## Ethical Statement

This research was approved by the Ethics Committee of Juntendo University (approval number E21-0080-H01). Since this was a retrospective observational study, there was no need to collect new samples from the patient. As a result, the need to obtain informed consent was waived.

## Informed consent statement

The study was opt-out for a retrospective study, so individual informed consent was waived.

## Data availability statement

Access to the information involved in the study is strictly limited by the Ethics Committee to protect patient privacy. Therefore, the dataset for this study is not publicly available. However, it may be obtained from the authors upon reasonable request, such as for non-commercial academic purposes.
